# A new GIS model for ecologically suitable distributions of medicinal plants

**DOI:** 10.1186/s13020-019-0226-0

**Published:** 2019-02-20

**Authors:** Jie Wu, Xiwen Li, Linfang Huang, Xiangxiao Meng, Haoyu Hu, Lu Luo, Shilin Chen

**Affiliations:** 1 Institute of Chinese Materia Medica, China Academy of Chinese Medical Sciences, Beijing, 100700 China; 2 Institute of Medicinal Plant Development, Chinese Academy of Medical Sciences and Peking Union Medical College, Beijing, 100193 China

**Keywords:** GMPGIS, Medicinal plant, Range-based method, Euclidean distance, Ecologically suitable regions

## Abstract

**Background:**

The endangered rate of medicinal plant exceeds that of endangered plant species. However, blindly introducing medicinal plants in regions without comprehensively considering the involved environmental factors results in diseases and insect pests and the consequent overproof pesticide residue as well as reduces the quality of herbal medicine produced.

**Methods:**

Global Medicinal Plant Geographic Information System (GMPGIS) was developed to analyze environmental information of ecologically suitable regions, thus guiding the conservation and introduction of medicinal plants. This system is based on theories and methods from multiple disciplines, including computer science, geoinformatics, ecology, and traditional herbal medicine. Using a range-based method, the previously established ecologically suitable regions were evaluated. This new method effectively resolved the problem of outlier points, and its functions were implemented in Python. The system automatically calculates the Euclidean distance of climatic factors and intersection of soil factors, thus identifying regions with high ecological similarity and those are climatically and edaphically suitable for the cultivation of medicinal plants.

**Results:**

These results, validated using real-world regions, revealed that GMPGIS is highly accurate in screening ecologically suitable regions for the cultivation of medicinal plants worldwide.

**Conclusions:**

Overall, because of these features, the GMPGIS is considered as a suitable distribution analysis system for global medicinal plant cultivation.

**Electronic supplementary material:**

The online version of this article (10.1186/s13020-019-0226-0) contains supplementary material, which is available to authorized users.

## Background

Since many years, analysing suitable regions for the cultivation of medicinal plants has been exclusively dependent on traditional experience, which is subject based on the observation of one or a few climatic factors. However, this method is inefficient and provides inaccurate results [1]. Thus, there is an unmet need to develop an effective method based on the geographical distribution of major producers and wild populations, reasonable analysis of climatic and soil conditions, and factors influencing medicinal plant growth. Such a method would aim to identify ecologically suitable regions worldwide, provide effective guidance for introducing medicinal plants, and develop a rational plan for geographically distributing plant cultivation [2, 3].

Adequate knowledge of ecologically suitable geographical regions is crucial for developing conservation and introduction strategies, but this information is extremely limited for most plant species. Geographic distribution models can be successfully used to extract information about ecological suitability and guide conservation and introduction efforts [4–7]. These models may be particularly helpful to exploit suitable planting areas in less well-known regions and for large-scale analyses because they allow the development of spatially comprehensive predictions of potential geographical distributions using incomplete information [8–10]. In recent years, these geographical distribution models have become one of the most frequently used approaches in macroecology and biodiversity conservation owing to the development of computer technology and the growth of the availability of environmental map layers and databases on different plant species.

The Global Medicinal Plant Geographic Information System (GMPGIS) was developed by the Institute of Chinese Materia Medica, China Academy of Chinese Medical Sciences. GMPGIS has the advantage of using presence-only data, and thus, it does not rely on or require data on confirmed absences from specific regions. The system contains a database of ecological environments that are beneficial for cultivating medicinal plants. The data are sourced primarily from two databases, namely WorldClim and Harmonized World Soil Database (HWSD), and provide consistent precision and coordinates for users. In addition, the following several aspects of the GMPGIS make it more practical than other geographical distribution models in predicting potential suitable regions of medicinal plant. Firstly, the system contains multiple sampling points and includes more than 240 medicinal plant species from geographical sampling points, which comprise the geographical distribution regions of both major producers and wild populations. Secondly, the system can extract values of ecological factors, soil categories and the area of potential growing regions using its built-in databases, to automatically generate tables for its users. Thirdly, the system avoids the outlier points when building an algorithm; therefore, the regions of which sampling points are included should present relatively high ecological similarity. Fourthly, the system avoids the problems arising from a small number of sampling points, particularly for species that are rare or have a restricted geographical range. With an insufficient number of sampling points, training algorithms frequently show poor performance and inaccuracies. Our system adopts an unsupervised learning analysis method to effectively solve these problems. A supervised verification method is also adopted to guarantee the accuracy of the analysis results. Finally, the system has already finished some basic operations, thereby simplifying and reducing the analysis processes for users. For instance, the number of ecological factors is reduced by principal component analysis (PCA). Furthermore, users can add 16 soil factors according to their requirements. Overall, because of these features, the GMPGIS is considered as a suitable distribution analysis system for global medicinal plant cultivation.

In the present study, the medicinal plant *Crocus sativus* L. (saffron crocus), which has been widely cultivated, was analysed as an example in detail using GMPGIS. Subsequently, we compared the system analysis results with the real-world regions of successful plant introduction. Moreover, the functions and methods of GMPGIS were also described.

## Materials and methods

### Study species

In the present study, *C. sativus* L. (saffron crocus) was adopted as an example to illustrate and verify the GMPGIS. Saffron is belongs to the Iridaceae family and is commercially available spice derived from the dried stigmas of the flower. The style of this plant also has medicinal uses, and it has very high economic value well known as ‘plant gold’. The dried stigmas of *C. sativus* L. have several applications, including promoting blood circulation, dissolving nodules and opening orifices and removing pathogenic heat from the blood and toxic materials from the body. Saffron crocus is also widely used in the perfume, food and dye industries. This plant is native to the Mediterranean, European and Asia Minor, and Western Asia regions, with Spain, France, Greece and Iran as the primary regions of cultivation. This species was first recorded in the *Ebers Papyrus* around 1550 AD and spread from India to Tibet [11–13]. In the early 1980s, saffron crocus was first planted in Chongming, Shanghai, China, where 90% of the country’s saffron is produced. In addition, the provinces of Jiangsu and Zhejiang also have saffron crocus plantations [14, 15].

In the proposed system, the point localities of the medicinal plants with the broadest geographic coverage were obtained from the Global Biodiversity Information Facility (http://www.gbif.org) and the Royal Botanic Gardens (http://www.kew.org). The geographical distribution points of both major producers and wild populations were used as the selection criteria and collated 158 occurrence records for this medicinal plant, originating primarily from Iran, France, Turkey, Greece, Germany, Italy, Sweden, Austria, Bulgaria, Syria and the United Kingdom.

### Environmental input variables

For the environmental data (version 2.0, representative of 1950–2000), we had no control over the risks arising from the use of hypothetical ecoclimatic factors as independent variables. Therefore, we adopted 19 bioclimatic factors as well as the average annual radiation and water vapour pressure from the original climatic factors in the WorldClim datasets downloaded from http://www.worldclim.org at a 30 s resolution (approximately 1 km^2^ spacing) [16, 17], thus totalling to 21 ecoclimatic factors. However, inputting a large dataset of relevant ecoclimatic covariates often lead to multicollinearity, a mathematical problem defined as a high degree of correlation amongst covariates in nonexperimental situations. PCA, a multivariate technique tool of ArcGIS 10.4, is used to reduce multiple data dimensions into a smaller number of analytical variables, which conserve a high proportion of the original information [18, 19]. This tool may be used to analyse the ecoclimatic variables in environmental layers. In the present study, PCA was performed to reduce the number of variables from 21 to 6 when some regions of polar climate were removed, such as the South Pole and North Pole. As a result, the data from these six factors were combined as the GMPGIS input data instead of the raw data of the ecoclimatic variables. In addition, soil data from HWSD version 1.1 were used as soil input variables (approximately 1-km^2^ spacing). These data are freely available at http://www.fao.org/soils-portal/soil-survey/soil-maps-and-databases/harmonized-world-soil-database-v12/en [20, 21]. We extracted 16 factors from the soil database according to the sampling points of the medicinal plants, adopting embedded Structured Query Language (SQL) produced from the suitable soil distribution layers. Details of the soil categories are presented (Table 1).

**Table 1:** Soil compositions

Field	Description	UNITS	DSMW	SOTWIS	CHINA	ESDB
T_GRAVEL	Topsoil gravel content	%vol.	√	√	√	√
T_SAND	Topsoil sand fraction	% wt	√	√	√	√
T_SILT	Topsoil silt fraction	% wt	√	√	√	√
T_CLAY	Topsoil clay fraction	% wt	√	√	√	√
T_USDA_TEX_CLASS	Topsoil USDA texture classification	Name	√	√	√	√
T_REF_BULK_DENSITY	Topsoil reference bulk density	kg/dm^3^	√	√	√	√
T_OC	Topsoil organic carbon	% weight	√	√	√	√
T_PH_H2O	Topsoil pH (H2O)	− log(H +)	√	√	√	√
T_CEC_CLAY	Topsoil CEC (clay)	cmol/kg	√	√	√	√
T_CEC_SOIL	Topsoil CEC (soil)	cmol/kg	√	√	√	√
T_BS	Topsoil base saturation	%	√	√	√	√
T_TEB	Topsoil TEB	cmol/kg	√	√	√	√
T_CACO3	Topsoil calcium carbonate	% weight	√	√	√	√
T_CASO4	Topsoil gypsum	% weight	√	√	√	√
T_ESP	Topsoil sodicity (ESP)	%	√	√	√	√
T_ECE	Topsoil SALINITY (Elco)	dS/m	√	√	√	√

### System principles and algorithm

Plant analysis was performed as follows. Firstly, we used PCA to reduce the ecological factor dimensions. Secondly, considering the influence of the different dimensions, all ecological factors were standardised. Thirdly, the algorithm was optimised using a range-based clustering method to analyse the potential distributions and render them suitable for evaluating the ecologically suitable regions. Fourthly, a clustering layer was reclassified, and the regions with maximum ecological similarity were acquired. Finally, the soil layers were intersected with the ecoclimatic factors in a Euclidean distance layer.

**Fig. 1:**
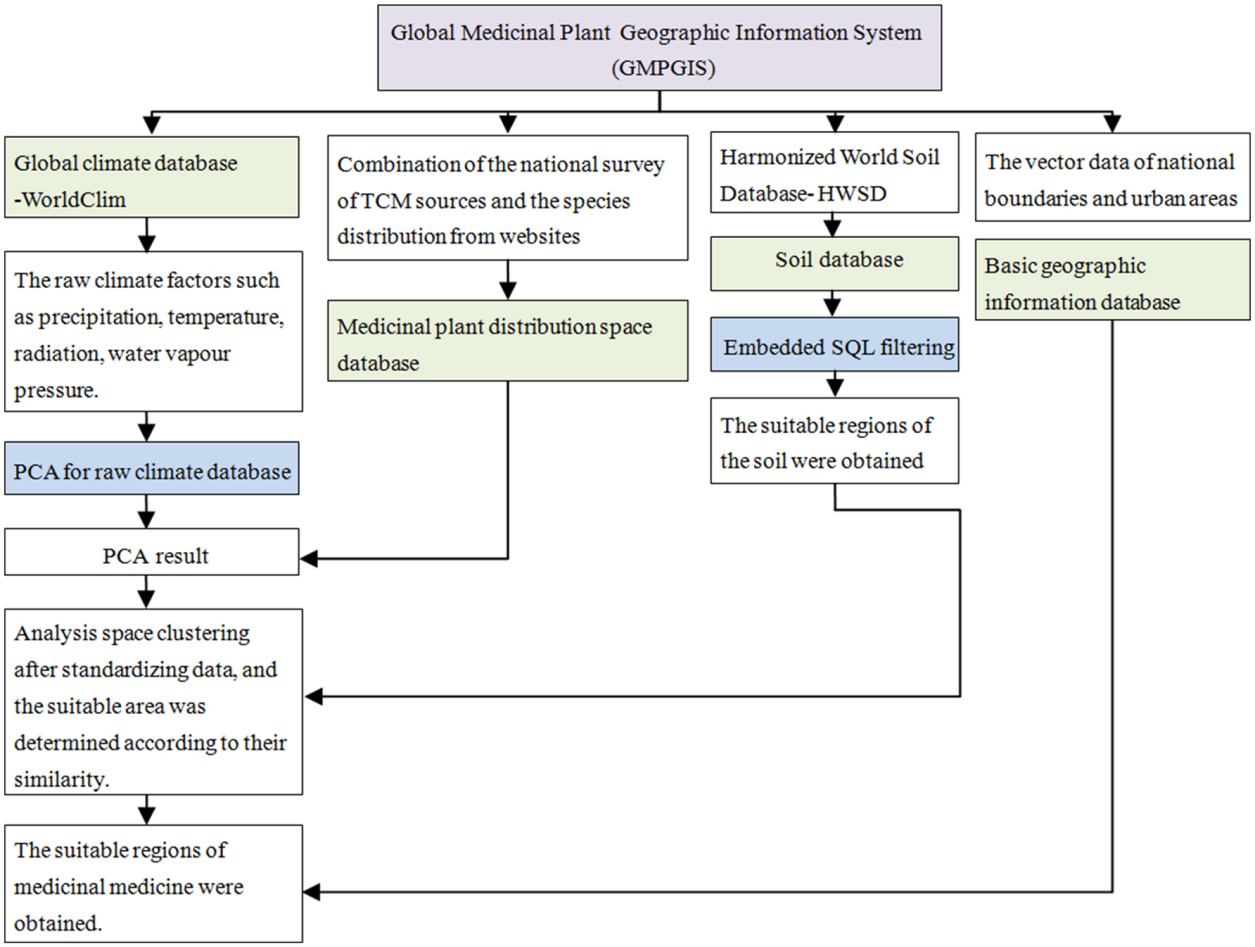
Working principle of the Global Medicinal Plant Geographic Information System (GMPGIS)

A range-based algorithm was adopted in this study. In some models, the individual sampling points or a small set of sampling points with ecological factor values that differed from those of the majority of the points were ignored in the running process. Nevertheless, we avoided the outlier problem using the GMPGIS and allowed every point to play a role in the analysis; that is, each region with sampling points had a similarity of 100% and was included in the suitable region. A range-based method was adopted to analyse the ecologically suitable regions and render them suitable for evaluating potential distributions. At present, range-based clustering has not been used in any other method. In addition, given that the lack of sample points adversely affects the accuracy of training, a non-training algorithm is more suitable for narrow habitat plants. The details of the analysis process are as follows (Fig. 1):

Step 1: PCA was used as a multivariate reduction algorithm of ArcGIS. Specifically, PCA was performed to reduce the number of variables from 21 to 6, and the results were selected as the GMPGIS input.

Step 2: Linear standardisation was performed on the PCA results. Supposing that *max*_*A*_ and *min*_*A*_ are the maximum and minimum values of layer A, respectively, the linear standardisation maps a value *v*_*i*_ from A to $v^{\prime}_{i}$ in the range [*newmin*_*A*_*, newmax*_*A*_] using the following calculation:

1 $$v_{i}^{'} = \frac{{v_{i} - min_{A} }}{{max_{A} - min_{A} }} \times 100$$

Step 3: An optimisation method was applied to analyse the ecologically suitable regions. A range-based partitioning algorithm used the critical value of a cluster (*D*_*i*_) on behalf of the cluster, defined as follows:

2 $$E = \mathop \sum \limits_{i = 1}^{k} \mathop \sum \limits_{{p \in D_{i} }}^{ } dist\left( {p,d_{i} } \right)^{2}$$

3 $${\text{S}}.{\text{t}}.\quad dist\left( {p,d_{i} } \right)=\left\{\begin{array} {l} 0, \quad \ newmin_{A} \le v_{i}^{'} \le newmax_{A} \\ min\left( {\left| {v_{i}^{'} - newmin_{A} } \right|,\left| {v_{i}^{'} - newmax_{A} } \right|} \right), \quad \ others \end{array} \right.$$

where *dist(p, d)* denotes the Euclidean distance between two points; *p* represents any point in the space, including the suitable and unsuitable distribution regions; *d*_*i*_ indicates the range of cluster *D*_*i*_ (both *p* and *d*_*i*_ are multidimensional); *newmin*_*A*_ is the minimum value after linearization of the layer and *newmax*_*A*_ is the maximum value after linearization of the layer (Additional file 1: Table S1).

Step 4: According to the calculation of the Euclidean distance [*Min*_*d*_, *Max*_*d*_], the raster was classified, and the potential ecological regions were obtained (Additional file 1: Table S2).

Step 5: The soil layer and the Euclidean distance layer of the ecological factors were overlapped through the following process: the raster points of the edaphically suitable regions were set to 1, and those of the unsuitable regions to 0. Similarly, the raster points of the ecologically suitable regions were set to 1, and those of the unsuitable regions to 0. These layers were intersected, and the raster points with values of 2 as the final result after adding these two layers together were extracted (Additional file 1: Table S3).

The Minimum Standards of Reporting Checklist contains details of the experimental design, and statistics, and resources used in this study (Additional file 2).

## Results

### Ecological factors of saffron crocus

Based on the sampling points of saffron crocus, the key threshold values of the ecological factors of the species were extracted from the GMPGIS (Table 2). The soil categories were primarily cambisols, leptosols, calcisols, luvisols, fluvisols and podzols, etc.

**Table 2:** Ecological factor ranges of *C. sativus* L

Biologically meaningful variables	Biological range	Biological mean	Biological Std Dev
BIO1 = annual mean temperature/°C	2.8–26.3	13.5	4.5
BIO2 = mean diurnal range (mean of monthly)/°C	5.6–16.7	11.3	2.8
BIO3 = isothermality (BIO2/BIO7) (*100)/°C	2.6–4.2	3.6	0.4
BIO4 = temperature seasonality (standard deviation * 100)	405.5–973.9	688.1	129.8
BIO5 = max temperature of warmest month/°C	15.9–42.7	30.2	6.0
BIO6 = min temperature of coldest month/°C	− 13.7–9.5	-6.0	4.5
BIO7 = temperature annual range (BIO5–BIO6)/°C	16.6–42.8	30.1	6.0
BIO8 = mean temperature of wettest quarter/°C	− 2.9–21.4	11.5	5.4
BIO9 = mean temperature of driest quarter/°C	0.8–34.3	19.5	8.8
BIO10 = mean temperature of warmest quarter/°C	11.1–35.3	22.4	5.0
BIO11 = mean temperature of coldest quarter/°C	2.8–26.3	13.5	4.5
BIO12 = annual precipitation/mm	58–1400	502.8	299.7
BIO13 = precipitation of wettest month/mm	13–226	67.2	32.6
BIO14 = precipitation of driest month/mm	0–93	19.2	22.8
BIO15 = precipitation seasonality (coefficient of variation)	8–124	47.5	28.5
BIO16 = precipitation of wettest quarter/mm	35–621	180.0	93.6
BIO17 = precipitation of driest quarter/mm	0–306	71.6	73.4
BIO18 = precipitation of warmest quarter/mm	0–321	86.5	86.1
BIO19 = precipitation of coldest quarter/mm	27–621	144.2	91.0
BIO20 = solar radiation/kJ m^−2^ day^−1^ (by calculation)	8628.6–19,770.5	15,603.9	2610.0
BIO21 = water vapor pressure/kPa (by calculation)	0.4–1.5	1.0	0.2

**Fig. 2:**
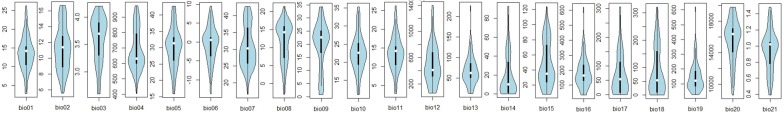
Violin plots of ecological factor ranges of the species

As shown in Table 1, the ecological factor values of saffron crocus vary considerably, but most cultivation of saffron crocus occurs primarily in Mediterranean, desert and semi-desert climate. The violin plots of the ranges of ecological factor values of the species are shown (Fig. 2).

### Analysis of the ecologically suitable regions of growing saffron crocus

**Fig. 3:**
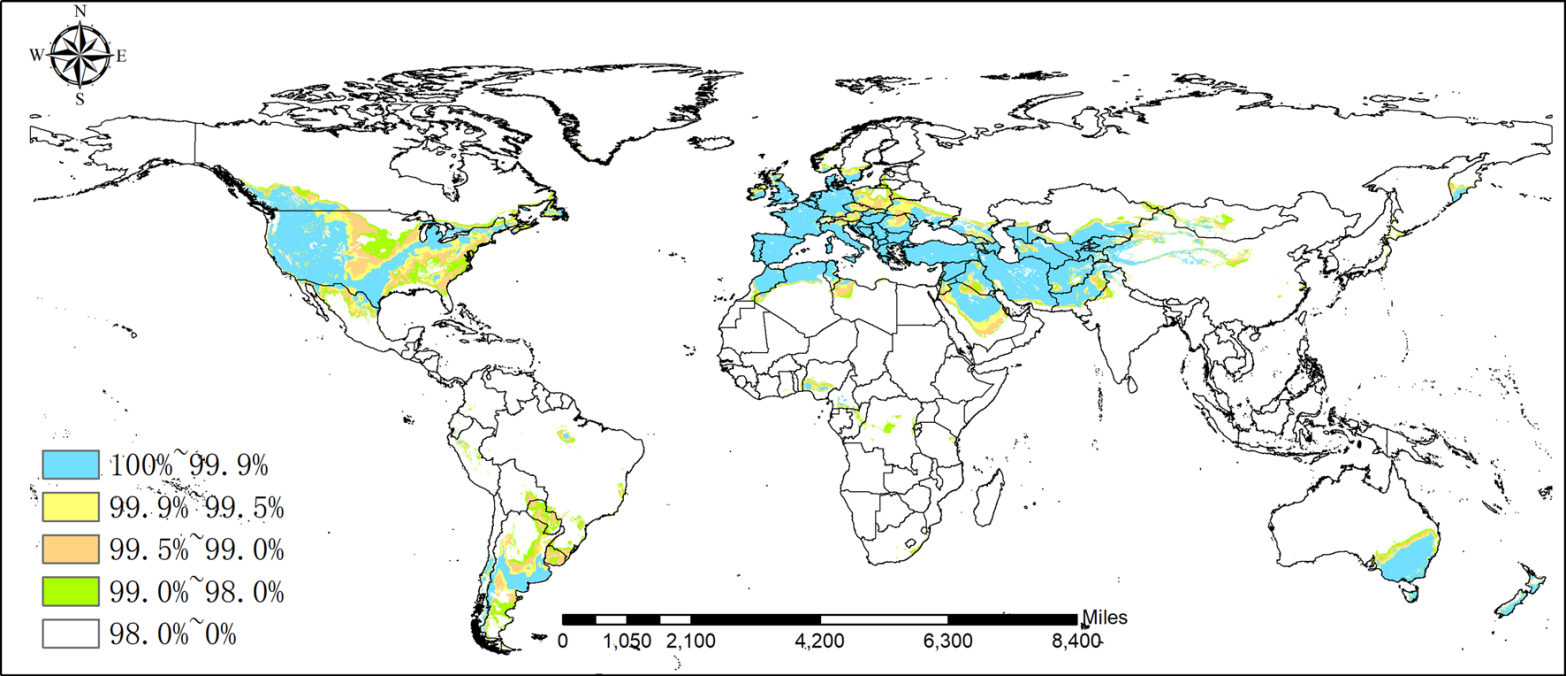
Potential distribution region analysis for *Crocus sativus* L. based on the Global Medicinal Plant Geographic Information System (GMPGIS) results. (Ecologically suitable distribution regions originated in ArcGIS 10.4, The GADM database of Global Administrative Areas 2.0 can be downloaded from http://www.gadm.org.)

**Fig. 4:**
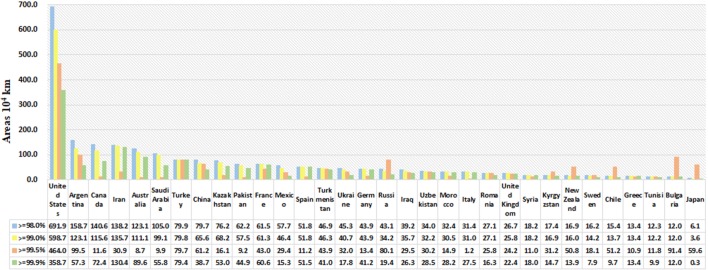
Areas with maximum ecological similarity of potential distribution primarily include the United States, Canada and Australia, etc

Saffron crocus is native to the Mediterranean, European and Asia Minor, and Western Asia regions, namely, it mainly grows in Spain, France, Turkey, Greece and Iran etc. The analysis shows that in addition to areas in the mentioned above, the United States and Canada in North America; China, Japan and India in Asia and Australia in Oceania also present a great introduction potential (Figs. 3 and 4).

### Validation and comparison results

**Fig. 5:**
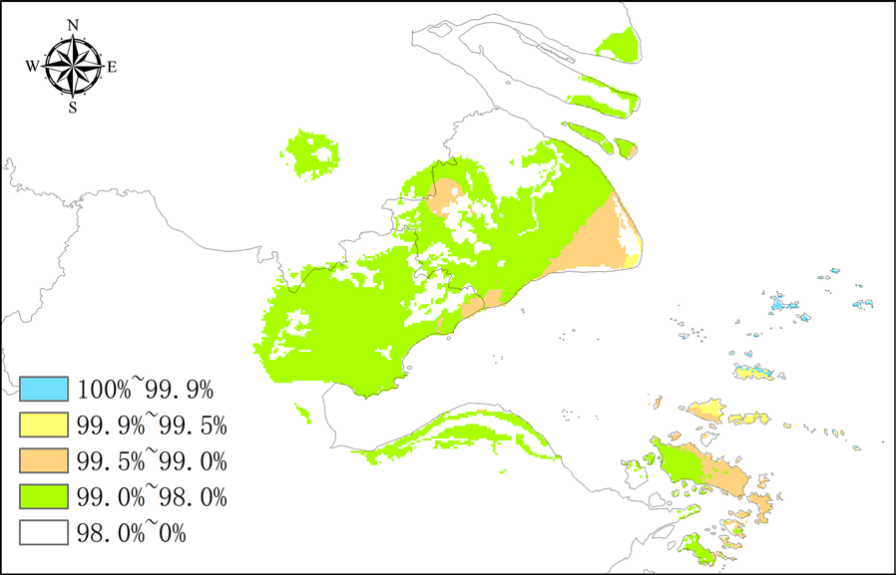
Analysis using GMPGIS showed that the Chongming Island in Shanghai, China is a potential region for introduction and distribution

**Fig. 6:**
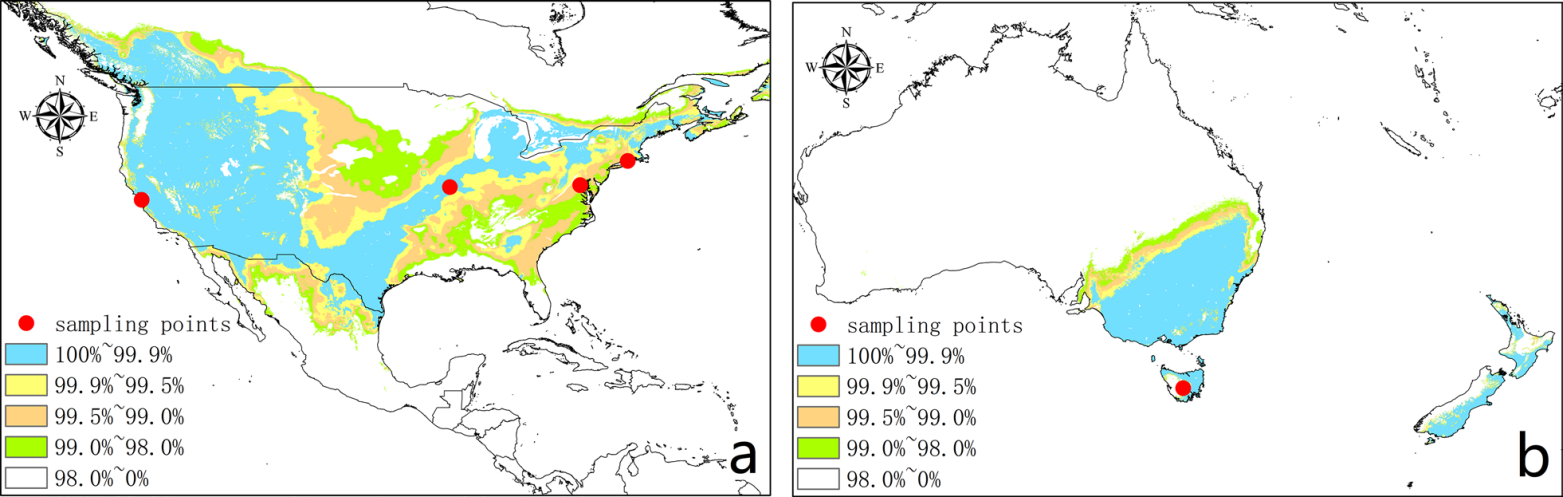
**a** Regions of successful introduction in California, Missouri, the District of Columbia and Connecticut, USA. **b** Other regions of successful introduction in Tasmania, Australia

Regions in which the saffron crocus has been successfully introduced were used as references to verify the results and validate the performance of GMPGIS. Using saffron crocus sampling points, which are primarily located in the Mediterranean, European and Asia Minor, and Western Asia regions, GMPGIS effectively analysed that the Chongming Island is a suitable region for introducing saffron crocus (Fig. 5) [14, 15], and these results were consistent with reality. In addition, successful introduction regions in the United States (http://www.gbif.org) (Fig. 6a) and Australia [22] (Fig. 6b) were included in the systematic analysis results. These results revealed that GMPGIS is highly accurate in screening ecologically suitable regions for the cultivation of medicinal plants worldwide.

## Discussion

### Strengths

We integrated the GMPGIS and the databases into a Python software package. The system is open source and is under continuous development (https://github.com/gmpgis/distribution). It features various functions of the GMPGIS: extraction of ecological factors, calculation of regions with maximum ecological similarity and calculation of suitable regions. In addition, the system also contains a sample point database and an ecological factor environment database, as well as a file with detailed user instructions. Currently, GMPGIS is being used to guide the introduction and cultivation of medicinal plant species such as *Acanthopanax senticosus* [23], *Paris polyphylla* var. *yunnanensis* [24], *Illicium* [25], *Panax* [26–28], *Taxus* [29] and *Dioscorea* [30]. Furthermore, the reliability of this approach has been comprehensively verified (Additional file 1: Table S4).

### Limitations

Although the GMPGIS was designed particularly to ensure ease of use, most GMPGIS users should be acquainted with Python, ArcGIS or other elementary operations. In addition, the system still lacks certain functions for many of the existing requirements (e.g. multiple system functions and sampling points in different parts of the world).

### Continued development

With the continuous expansion of introduction regions for Chinese medicinal plants, the GMPGIS will continuously adjust the sampling data and incorporate additional wild geographical distribution and major production regions into the database. The regions where medicinal plants have been successfully introduced may be used as references to verify and improve the accuracy of the system and optimise the analysis results. In addition, the GMPGIS is capable of establishing a more convenient and efficient web server that will allow different researchers to rapidly obtain accurate analysis results.

## Conclusions

China has a vast territory, and its different regions present distinct variations in climate and soil. Likewise, the quality of the medicinal plant materials of the same species growing in different ecological environments is different, and this is especially true for Chinese medicinal plant materials, the cultivation and effects of which are strongly influenced by ecological factors. The quality of medicinal materials in different production regions is superior when the region is highly similar to the regions where Daodi medicinal materials are produced. Based on this premise and the ecological similarity principle, a reasonable plan for the introduction of Chinese medicinal material can be designed, and various regions for planting Chinese medicinal materials can be developed. Continuous cropping obstacle is a primary reason for decreases in medicinal material yield, poor planting quality and aggravation of disease and insect pests. As the planting region continues to reduce, selecting new lands for cultivation is necessary to guarantee the development of high-quality medicinal materials. The scientific analysis of potentially suitable production regions should be conducted using the GMPGIS for reasonable migration and base construction [29, 31, 32].

This study found that good consistency exists between the maximum ecological similarity analysis results obtained using the GMPGIS and the ‘Belt and Road Initiative’ advocated by China. The geographical regions included in this initiative, namely Russia, the Netherlands, Germany, Italy and Turkey, have high-potential production regions. With continuous advancements in agricultural cultivation technology, this study may greatly promote the internationalisation of the Chinese herbal medicine planting industry [27].

## Additional files

**Additional file 1: Table S1.** Calculation of Euclidean distance. **Table S2.** Classification of raster. **Table S3.** Calculation of suitable soil. **Table S4.** Summary of published species.

**Additional file 2.** Minimum Standards of Reporting Checklist.

## Abbreviations

GMPGIS: Global Medicinal Plant Geographic Information System
