# Supplementary material for: A new GIS model for ecologically suitable distributions of medicinal plants
Source: Chin Med. 2019 Feb 20;14:4. doi: 10.1186/s13020-019-0226-0 (PMC6383245; doi:10.1186/s13020-019-0226-0)
Supplement: Supplementary file 1 — Additional file 1: Table S1. Calculation of Euclidean distance. Table S2. Classification of raster. Table S3. Calculation of suitable soil. Table S4. Summary of published species. [file 13020_2019_226_MOESM1_ESM.docx]

**Additional Tables**

**Table S1**. Calculation of Euclidean distance

| **Algorithm 1** |
| --- |
| Raster=Con((envRaster>=min) & (envRaster <=max),0,Con(envRaster >=(max+min)//2,max-envRaster,envRaster-min))  Raster.save(outPut+"/"+listName)  PC1 = Raster(outPut+"/ PC1")  PC2 = Raster(outPut+"/ PC2")  PC3 = Raster(outPut+"/ PC3 ")  PC4 = Raster(outPut+"/ PC4")  PC5 = Raster(outPut+"/ PC5 ")  PC6 = Raster(outPut+"/ PC6")  Eu_dis =( PC1 * PC1 * w_1_ + PC2 * PC2 * w_2_ + PC3 * PC3 * w_3_ + PC4 * PC4 * w_4_ + PC5 * PC5 * w_5_+ PC6 * PC6* w_6_ )**0.5  Eu_dis.save(outPut+"/Eu_dis") |

**Table S2**. Classification of raster

| **Algorithm 2** |
| --- |
| Re_map = RemapRange([[0,0.1,1],[0.1,100,2]])  Re_edis = Reclassify(dis,"Value", Re_map)  Re_edis.save(outPut+"/Re_edis") |

**Table S3**. Calculation of suitable soil

| **Algorithm 3** |
| --- |
| for num in T_USDA_TEX_CLASS:  if num not in inlist:  inlist.append(num)  inlist.sort()  max=inlist[-1]  for num in inlist:  if num>=max:  sql=sql+"HWSD_DATA.T_USDA_TEX_CLASS={0}".format(num)  break;  else:  sql=sql+"HWSD_DATA.T_USDA_TEX_CLASS={0} OR ".format(num)  sql=sql+"AND HWSD_DATA.T_GRAVEL >= {0} AND T_GRAVEL <= {1}".format(T_GRAVEL_MIN,T_GRAVEL_MAX)  sql=sql+"AND HWSD_DATA.T_SILT >= {0} AND HWSD_DATA.T_SILT <= {1}".format(T_SILT_MIN,T_SILT_MAX)  sql=sql+"AND HWSD_DATA.T_CLAY >={0} AND HWSD_DATA.T_CLAY <={1}".format(T_CLAY_MIN,T_CLAY_MAX)  sql=sql+"AND HWSD_DATA.T_REF_BULK_DENSITY >={0} AND HWSD_DATA.T_REF_BULK_DENSITY <={1}".format(T_REF_BULK_MIN,T_REF_BULK_MAX)  sql=sql+"AND HWSD_DATA.T_BULK_DENSITY >={0} AND HWSD_DATA.T_BULK_DENSITY <={1}".format(T_BULK_DEN_MIN,T_BULK_DEN_MAX)  sql=sql+"AND HWSD_DATA.T_OC >={0} AND HWSD_DATA.T_OC <={1}".format(T_OC_MIN,T_OC_MAX)  sql=sql+"AND HWSD_DATA.T_PH_H2O >={0} AND HWSD_DATA.T_PH_H2O <={1}".format(T_PH_H2O_MIN,T_PH_H2O_MAX)  sql=sql+"AND HWSD_DATA.T_CEC_CLAY >={0} AND HWSD_DATA.T_CEC_CLAY <={1}".format(T_CACO3_MIN,T_CACO3_MAX)  sql=sql+"AND HWSD_DATA.T_CEC_SOIL >={0} AND HWSD_DATA.T_CEC_SOIL <={1}".format(T_CEC_SOIL_MIN,T_CEC_SOIL_MAX)  sql=sql+"AND HWSD_DATA.T_BS >={0} AND HWSD_DATA.T_BS <={1}".format(T_BS_MIN,T_BS_MAX)  sql=sql+"AND HWSD_DATA.T_TEB >={0} AND HWSD_DATA.T_TEB <={1}".format(T_TEB_MIN,T_TEB_MAX)  sql=sql+"AND HWSD_DATA.T_CACO3 >={0} AND HWSD_DATA.T_CACO3 <={1}".format(T_CACO3_MIN,T_CACO3_MAX)  sql=sql+"AND HWSD_DATA.T_CASO4 >={0} AND HWSD_DATA.T_CASO4 <={1}".format(T_CASO4_MIN,T_CASO4_MAX)  sql=sql+"AND HWSD_DATA.T_ESP >={0} AND HWSD_DATA.T_ESP <={1}".format(T_ESP_MIN,T_ESP_MAX)  sql=sql+"AND HWSD_DATA.T_ECE >={0} AND HWSD_DATA.T_ECE <={1}".format(T_ECE_MIN,T_ECE_MAX)  soilRaster=Con("hwsd",10,0,sql) |

**Table S4**. Summary of published species

| **Species** | **Original production regions** | **Potential distribution regions** |
| --- | --- | --- |
| Published in China J. Chin. Mater. Med., 2016 | | |
| *Panax ginseng* | China: Northeast China  Globally: North Korea, South Korea, Japan, Russia and the Far-East. | Russia, France, Italy, Ukraine, Serbia, Bulgaria, Spain, Hungary, Romania, Germany, Switzerland in Europe, the United States and Canada in North America, etc. |
| Published in Acta Pharm. Sin., 2016 | | |
| *Panax notoginseng* | China: Southwest | Sporadic distribution covers the east coast of the United States in North America; the southeast of Brazil in South America; Portugal, Spain, and France on the west coast of Europe; and Italy along the northern Mediterranean area. |
| Published in Chin. J. Exp. Tradit. Med. Form., 2017 | | |
| *Paris polyphylla* var. *yunnanensis* | China: Yunnan, Sichuan, Guizhou, Hunan, Guangxi and Chongqing provinces.  Globally: Nepal, Bhutan, Sikkim, Vietnam, Thailand, Myanmar, and Laos. | The United States in North America, Brazil in South America; India in Asia; and Zambia, Congo, and Tanzania in Africa, etc. |
| Published in Acta Pharm. Sin., 2017 | | |
| *Taxus wallichiana* var. *chinensis* | China: Gansu, Shaanxi, Sichuan, Yunnan, Guizhou, Hubei, Hunan, Guangxi and Anhui provinces. | The United States, Argentina and Brazil have great potential as distribution regions; Spain, Tanzania, Mexico, France, Russia, and Turkey have a potential for development in introduction and cultivation. |
| *Taxus baccata* | Globally: Caucasus area, from eastern Turkey to northern Iran in Europe, Morocco, and Algeria in northern Africa and Iraq in southwest Asia. | The United States, China and Russia have great potential as distribution regions. Brazil, Argentina, Canada, Australia, Mexico and Turkey have great development potential. |
| *Taxus floridana* | Globally: Northwest Florida in the United States. | Only a small area (10 km^2^) on the eastern side of the Apalachicola river at an elevation of 15~30 m in north Florida and the United States. |
| Published in Mol., 2017 | | |
| *Panax japonicus* | China: Yunnan, Guizhou, Sichuan, and Hubei  Globally: Japan and North Korea. | Southeast Asian and North American countries such as China, Japan, South Korea, North Korea, the United States and Canada. |
| *Panax japonicus* var.*major* | China: Shaanxi, Sichuan, Yunnan, Gansu, and Guizhou provinces. | North America, south Asia, and Europe (Italy, Spain, French and Russia). |
| *Panax zingiberensis* | China: Yunnan province  Globally: Burma | Some sporadic regions surrounding the South of Brazil, other parts of Asia and South America. |
| *Panax stipuleanatus* | China: Yunnan province  Globally: Burma | Some sporadic regions surrounding the South of Brazil and other parts of Asia. |
| Published in Chin. J., 2018 | | |
| *Illicium verum* | China: Guangxi, Jiangxi, Chongqing, Hainan, Zhejiang, Hunan, Fujian, Guangdong, Guizhou, and Yunnan provinces. | The species is also found in the United States and Brazil. |
| *Illicium henryi* | China: Jiangxi, Henan, Guangdong, Shanxi, Chongqing, Fujian, Anhui, Guangxi, Yunnan, Gansu, Hubei, Sichuan and Guizhou provinces. | The species additionally has other suitable habitats in the United States (the second-largest coverage region). It also thrives in France and Italy. |
| *Illicium majus* | China: Guangxi, Sichuan, Guizhou, Chongqing, Yunnan, Guangdong, Hunan, and Hubei provinces.  Globally: Southern Burma and northern Vietnam. | The species is also found in other potential distribution regions in the United States, Brazil and Japan, etc. |
| Published in Acta. Pharm. Sin., 2018 | | |
| *Acanthopanax senticosus* | China: Lesser Khingan, Changbai mountains, Great Khingan, Yanshan Mountains, Taihang mountains, and Qinling mountains. | Near areas of great lakes in the northwest of the United States and southwest of Canada; the regions of eastern and central Europe, such as Ukraine, Romania, Hungary, and Germany, southwest Russia; and the border between Kazakhstan and China’s Sinkiang area in the central Asian inland. |
| Published in Chin. J., 2018 | | |
| *Dioscorea althaeoides* | China: Southwest part of Yunnan, Sichuan, Guizhou and Tibet, central region of Shanxi, Chongqing and Hunan, southern part of Zhejiang and Jiangxi provinces. | The United States, France, Russia, Italy, Ukraine, Brazil, Australian, Spain, Rumania, New Zealand and Bulgaria, etc. |
| *Dioscorea collettii* | China: Sichuan, Yunnan, Guizhou, Chongqing, Guangxi, Hunan, Taiwan and Shanxi provinces.  Globally: Japan’s Honshu. | The United States, Brazil, The republic of Congo, Burma, Peru, Bolivia, Mexico, Thailand, Tanzania and Angola, etc. |
| *Dioscorea deltoidea* | China: Sichuan, Yunnan, Guizhou, Tibet and Taiwan provinces.  Globally: Northern and central part of India, Sikkim, Bhutan, Nepal, northeast part of Pakistan, few parts of [Afghanistan](javascript:void(0);), southern part of Thailand and central region of Laos. | The United States, Brazil, Australian, India, the republic of Congo, Kazakhstan, Mexico, Bolivia, South Africa, Argentina and Turkey, etc. |
